# Developing a morphomics framework to optimize implant site-specific design parameters for islet macroencapsulation devices

**DOI:** 10.1098/rsif.2021.0673

**Published:** 2021-12-22

**Authors:** Barry McDermott, Scott Robinson, Sven Holcombe, Ruth E. Levey, Peter Dockery, Paul Johnson, Stewart Wang, Eimear B. Dolan, Garry P. Duffy

**Affiliations:** ^1^ Translational Medical Device Lab, College of Medicine Nursing and Health Sciences, National University of Ireland (NUI) Galway, Galway, Ireland; ^2^ Anatomy and Regenerative Medicine Institute (REMEDI), School of Medicine, College of Medicine Nursing and Health Sciences, National University of Ireland (NUI) Galway, Galway, Ireland; ^3^ Biomedical Engineering, School of Engineering, College of Science and Engineering, National University of Ireland (NUI) Galway, Galway, Ireland; ^4^ CURAM, Centre for Research in Medical Devices, National University of Ireland (NUI) Galway, Galway, Ireland; ^5^ Section of Vascular Surgery, Department of Surgery, University of Michigan, Ann Arbor, MI, USA; ^6^ Nuffield Department of Surgical Sciences and NIHR Biomedical Research Centre, Oxford Centre for Diabetes Endocrinology and Metabolism, University of Oxford, Oxford, UK; ^7^ Advanced Materials and BioEngineering Research Centre (AMBER), Royal College of Surgeons in Ireland and Trinity College Dublin, Dublin, Ireland

**Keywords:** macroencapsulation device, morphomics, islet transplantation, beta-cell transplantation, implant site

## Abstract

Delivering a clinically impactful cell number is a major design challenge for cell macroencapsulation devices for Type 1 diabetes. It is important to understand the transplant site anatomy to design a device that is practical and that can achieve a sufficient cell dose. We identify the posterior rectus sheath plane as a potential implant site as it is easily accessible, can facilitate longitudinal monitoring of transplants, and can provide nutritive support for cell survival. We have investigated this space using morphomics across a representative patient cohort (642 participants) and have analysed the data in terms of gender, age and BMI. We used a shape optimization process to maximize the volume and identified that elliptical devices achieve a clinically impactful cell dose while meeting device manufacture and delivery requirements. This morphomics framework has the potential to significantly influence the design of future macroencapsulation devices to better suit the needs of patients.

## 1. Background

Type 1 diabetes (T1D) is a global problem affecting 18 million people. It is characterized by autoimmune destruction of insulin-producing β-cells within pancreatic islets and results in lifelong inability to regulate blood glucose levels. Conventional treatment involves delivery of exogenous subcutaneous insulin via injection intermittently or by a pump. Besides the life-altering treatment burden, this strategy can also result in sub-optimal glycaemic control leading to severe complications and death [1]. Donor pancreatic islet transplantation has gained attention as it has the potential to re-establish naturally regulated insulin production [2–4]. The most widely used strategy involves infusion of isolated cadaveric islets into the liver via the portal vein and administration of systemic immunosuppression. A recent report of the Collaborative Islet Transplant Registry shows that 5-years following islet transplantation, greater than 90% of patients achieve avoidance of severe hypoglycaemic episodes, approximately 60% achieve optimal glycaemic control (HbA1c < 7%), and approximately 30% achieve insulin independence [5]. Ten-year outcomes have begun to appear and show optimal glycaemic control and insulin independence rates of only 18–28% [6,7]. It is widely accepted that early islet cell death and lack of long-term graft survival due to alloimmune and autoimmune rejections have prevented sustained therapeutic effects [2,3,8–10]. Traditional islet transplantation via infusion through the portal vein is now recognized as a major contributor to transplanted cell death, where it is estimated that 50–70% of transplanted cells are lost prior to engraftment in the liver [11,12]. This observation combined with the associated side-effects of lifelong immunosuppression, has led research to focus on more favourable extra-hepatic engraftment sites where cells are enclosed in a macroencapsulation device that contains a semipermeable barrier providing immunoprotection of transplanted cells while allowing free diffusion of glucose and insulin [13–18]. Macroencapsulation devices hold promise, but a number of challenges still need to be overcome [13], such as the limited availability of donor cells, the determination of the optimal implant site and the optimization of the size, design and shape of the devices.

Limited availability of functional donor pancreases has prevented widespread implementation of islet transplantation since two or more donor pancreases are often needed to achieve enough islets to reverse diabetes (10 000 IEQ kg^−1^) [2]. While islet isolation methods have improved, advances in the development of unlimited sources of insulin-producing cells [19–22] hold realistic promise for the future expansion of islet/β-cell transplant therapy. ViaCyte’s PEC-01 cells [19,23] and Vertex Pharmaceuticals Incorporated VX-880 cells are currently the only stem cell derived islet cell replacement therapies in clinical trials for T1D.” This activity demonstrates a transition to off-the-shelf cell sources, but the challenge of implant and delivery remain the same. To fully realize the benefits of islet encapsulation, an appropriate implant site must be identified. Such a site must be clinically accessible via a low-risk procedure, enable long-term monitoring of islet function, enable retrievability in the event of a complication and provide a suitable environment for the safeguarding of efficacious islet function. A number of potential implant sites have been discussed, such as the subcutaneous [24–26] and submuscular spaces [27], the peritoneum [28,29] and the omentum [30]. Currently, there has not been consensus on the optimal anatomical location. Interestingly, the primary focus of Viacyte's ongoing clinical trial (NCT02239354), in addition to demonstrating safety and tolerability, is to understand factors affecting implanted cell survival, including the nature and intensity of host response, surgical implantation procedures, anatomical location and peri-operative care.

The size and shape of macroencapsulation devices are critically important for islet/β-cell transplantation. Achieving a sufficient cell dose is a major design challenge as a high cell number is required to reverse T1D, while issues associated with cell overcrowding must be overcome. Islet density has been recommended to be 5–10% of the volume fraction [14,31]. Additionally, cells should not be greater than 0.3 mm from the porous membrane of immune isolation macroencapsulation devices, where the ingrowth of vasculature is inhibited, to allow sufficient transport of vital oxygen and nutrients. Device shape significantly affects the distribution of the interfacial forces where higher stress concentrates at sharp angles, curves and edges, have been shown to induce a strong foreign body response [13,32]. A survey of 482 people with T1D investigating their preferences on the size, shape, visibility and transplantation site of islet containing implants showed that 52.7% of people preferred the location to be under the skin and 58.4% preferred the implant to be as small as possible [33]. Macroencapsulation devices have emerged in various sizes and shapes, with some reaching clinical trials including β-Air by Beta-O_2_ Technologies Ltd and PEC-Encap (VC-01) by ViaCyte. In these trials, Beta-O_2_ Technologies are using allogenic human islets as the cell source and have used 1800–4600 IEQ kg^−1^ [15]. Viacyte's devices are rectangular, similar in size and shape to a credit card, while β-Air are circular. To achieve a sufficient cell dose to reverse T1D, the approach of implanting multiple devices has been taken by ViaCyte where 2–6 PEC-Encap devices are implanted per patient (NCT02239354) and Beta-O_2_ where 1–2 β-Air devices are implanted per patient [15].

The posterior rectus sheath plane (PRSP) represents a potential implant site that could support a cell-containing implant. This plane lies between the muscle belly of the rectus abdominus and the fascia of the rectus abdominus muscle. There is precedent for accessing this space in a minimally invasive fashion as it is often accessed using ultrasound to provide analgesia for abdominal surgery [34]. The superficial location of the site allows for longitudinal monitoring with non-invasive imaging, and access for graft biopsy if needed. Implantation and retrieval can be performed without violating the peritoneal space, thereby avoiding the numerous complications associated with intra-abdominal procedures. Due to the bilateral location of the site, it can provide two distinct implant sites that do not directly communicate. Differences in site from left to right is important when planning strategies that may require two interventions such as islet transplantation. This site has a robust blood supply, with contributions from the superior and inferior epigastric arteries branching from the internal thoracic and external iliac arteries, respectively, with additional small tributaries of the lower six internal intercostal arteries [35,36]. This dense vascular network could permit rapid development and distribution of neovascularization adjacent to the macroencapsulation device surface thus ensuring that encapsulated cells would receive adequate oxygen and nutrient supply for survival and function [37]. Thus, the potential space within the posterior rectus sheath meets the requirements for being easily accessible, facilitates longitudinal monitoring of transplants and provides nutritive support for islet cell survival. It is important to understand the anatomy of the PRSP across a target patient population to design a device that can achieve a clinically impactful cell dose.

In this study, we used human imaging and morphomics analysis to describe a novel implant site for macroencapsulation devices. Using geometric data obtained from a reference population of computed tomography (CT) scans, we employed a shape optimization process to inform device design to maximize the number of transplanted islets. Quantitative and categorical variables and anatomical measurements were used as features in machine-learning regression algorithms. The regression model predicts the average area of the maximal fitted devices for each patient. A classification analysis predicts theoretical off-the-shelf devices for each shape. These algorithms could be used as a screening tool prior to extensive imaging to allow for patient stratification and pre-intervention planning. We demonstrate how a morphomics approach can guide device design, for both a patient-specific approach and a target patient population approach. This information has the potential to significantly impact the field and may influence the design of macroencapsulation devices.

## 2. Results

### 2.1. Morphomics characterization of the posterior rectus sheath

**Figure 1.**
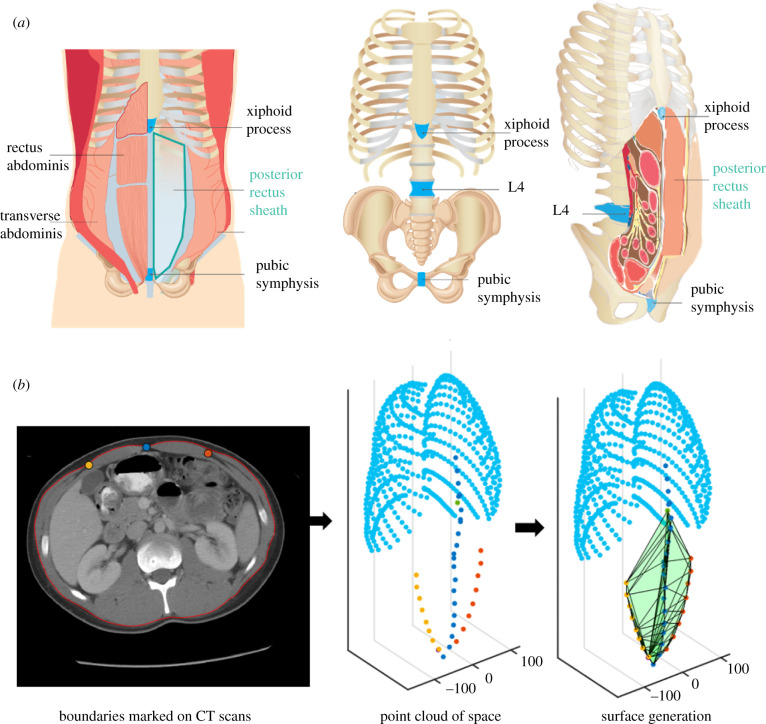
Graphical representation of the PRSP and analysis used in this study. (*a*) Schematic of anatomy of the location of the PRSP enclosed by the xyphoid process (cranially), tip of the pubic symphysis (caudally), linea alba (medially) and semilunar line points (laterally). (*b*) Arbitrary participant showing the points from the segmentation from CT scans and the resulting computed space.

A total of 642 participants were included in this study, 304 of which were female (47.35%). Patient characteristics are summarized in table 1. A series of semi-automated segmentation algorithms were used to identify anatomical boundaries within each scan that border the PRSP to identify a potential space within the posterior rectus sheath, figure 1*a*. Imaging data were used to specify anatomical positions with Cartesian coordinates in *R*^3^ (figure 1*b*) including points along all ribs, the caudal tip of the xyphoid process, the cranial aspect of the pubic symphysis and points along the bilateral semilunar lines. Each set of points was next processed using MATLAB to define computational models of specific geometry in Cartesian coordinates for each patient (figure 1*b*). The PRSP was defined as the potential space enclosed by the xyphoid process (cranially), tip of the pubic symphysis (caudally), linea alba (medially) and semilunar points (laterally). The PRSP was thus defined on both the left and right sides, with both planes sharing the xyphoid process, tip of the pubic symphysis and linea alba. Upon computation of the three-dimensional boundary of the space, the volume of each PRSP could be calculated for each patient. Maximal fitting circular, rectangular, polygonal and elliptical shapes were compared across age, gender and BMI.

**Table 1.** Summary of patient characteristics. Patient characteristics at date of CT by sex. “a” indicates the measurement was taken from CT scans and “b” indicates the measurement was taken at the level of the umbilicus assumed to be at L4. Unpaired Mann–Whitney (not normally distributed Shapiro–Wilk normality test).

	females		males		*p*
	*N*	mean ± s.d.	*N*	mean ± s.d.	
age (years)	304	34.36 ± 23.94	338	31.02 ± 20.84	0.1711
BMI (kg m^−2^)	266	26.3 ± 7.51	300	26.33 ± 6.90	0.7928
height (m)	267	1.59 ± 0.15	302	1.72 ± 0.20	<0.0001
weight (kg)	299	64.79 ± 25.37	330	76.97 ± 28.97	<0.0001
distance between the left and right anterior superior iliac spine (ASIS)^a^	305	213.67 ± 31.52	338	219.66 ± 32.80	0.0070
distance from xyphoid process to the centre of the pubic symphysis^a^	302	53.41 ± 17.79	331	72.30 ± 24.24	<0.0001
body depth^a^^b^	302	216.69 ± 54.74	331	222.35 ± 58.66	0.3412
body width^a^^b^	302	315.84 ± 62.56	331	314.41 ± 61.20	0.4977
body circumference^a^^b^	302	878.93 ± 192.70	331	880.89 ± 198.36	0.8814

^a^Measured from CT scans. ^b^Measurement taken at the level of the umbilicus assumed to be at L4.

From within each scan, a series of anatomical parameters was captured that could be easily measured on physical examination through identification of surface landmarks. These included distance between the left and right anterior superior iliac spine, distance from xyphoid process to the centre of the pubic symphysis, body depth, body width and body circumference (the latter three were measured at the level of L4 which was found to lie nearest the umbilicus), as shown in figure 1*a* and patient measurements are summarized in table 1.

**Figure 2.**
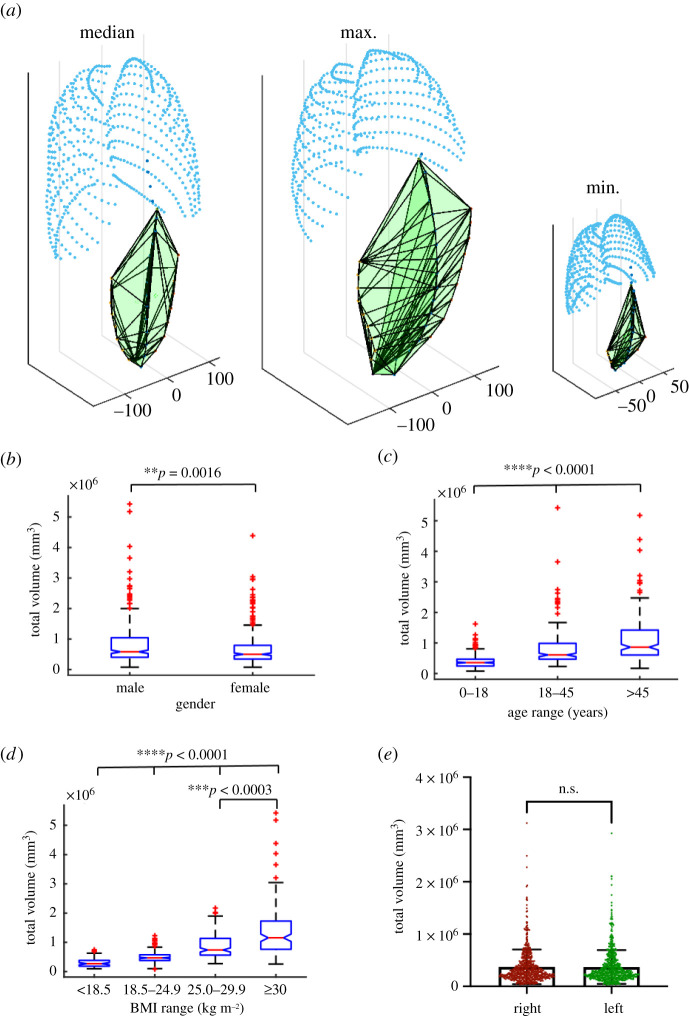
Analysis of the available volume of the PRSP. Graphical representation of the volume of the PRSP (shown in green) and rib cage (shown in aqua) for the median, maximum and minimum participants (*a*). Analysis of the total volume of the PRSP with respect to gender (*b*), age (*c*) and BMI (*d*). Analysis of the volume on the left versus right side of the linea alba (*e*). Data are means ± s.d.; **p < 0.01, ***p < 0.001, ****p < 0.0001, n.s., not significant.

The volume of the PRSP is shown in figure 2*a* where the median, maximum and minimum geometries are shown. PRSP volume was significantly smaller in females (male = 0.81 ± 0.68 × 10^6^ versus female = 0.68 ± 0.57 × 10^6^ mm^3^, **p = 0.002, figure 2*b*). All age groups were significantly different to each other (0–18 = 0.39 ± 0.22 × 10^6^ versus 18–45 = 0.81 ± 0.60 × 10^6^ versus >45 = 1.13 ± 0.77 × 10^6^ mm^3^, ****p < 0.001, figure 2*c*). When stratified according to BMI, underweight and normal weight patients had a PRSP volume that was statistically different to all other groups (18.5 = 0.297 ± 0.16 × 10^6^ versus 18.5–24.9 = 0.495 ± 0.19 × 10^6^ versus 25–29.9 = 0.86 ± 0.40 × 10^6^ versus >30 = 1.39 ± 0.92 × 10^6^ mm^3^, ****p = 0.001), while overweight participants had a statistically smaller volume than obese patients (25–29.9 = 0.86 ± 0.40 × 10^6^ versus >30 = 1.39 ± 0.92 × 10^6^ mm^3^, ***p < 0.001, figure 2*d*). There was no statistical difference in PRSP volume of each side (right = 0.37 ± 0.33 × 10^6^ versus left = 0.37 ± 0.32 mm^3^, figure 2*e*).

### 2.2. Design of patient-specific polygonal devices in the PRSP

**Figure 3.**
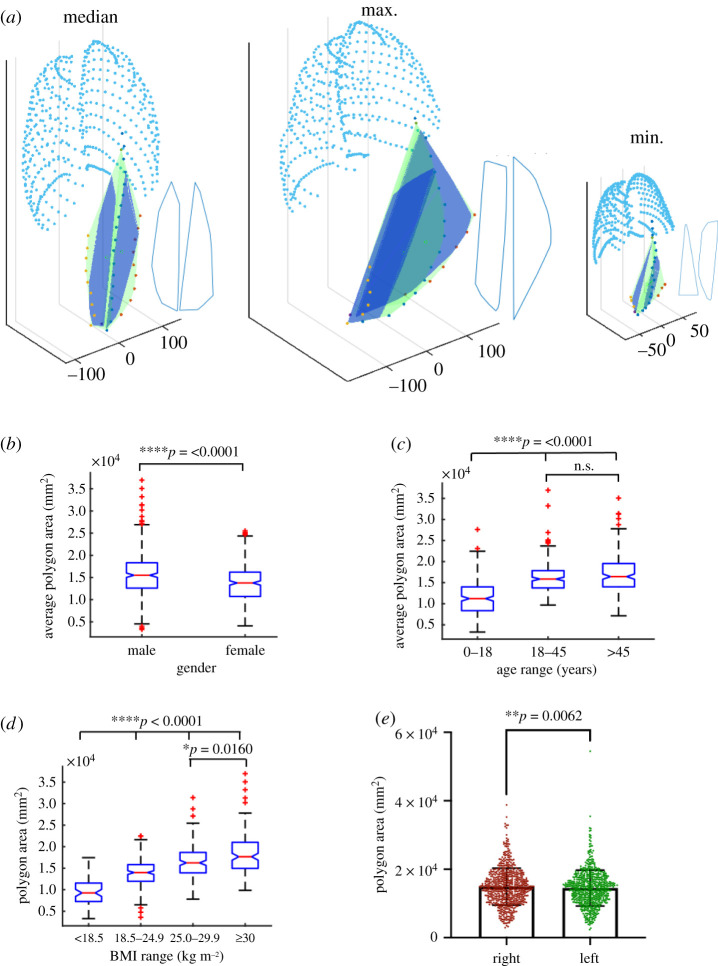
Analysis of the maximum fitted polygon in the PRSP. Graphical representation of the area of the maximum fitted polygon (shown in royal blue) in the PRSP (shown in green) and ribs (shown in aqua) for the median, max and minimum participants. Outline of the shape is also shown in blue beside each (*a*). Analysis of the average area of the maximum fitted polygon in the PRSP with respect to gender (*b*), age (*c*) and BMI (*d*). Analysis of the area of the maximum fitted polygon on the left versus right side of the linea alba (*e*). Data are means ± s.d.; *p < 0.05, **p < 0.01, ***p < 0.001, n.s. = not significant.

To use as much of the available space in the PRSP as possible, the maximum-sized polygonal-shaped devices were fitted in the space. For our analyses, we determined the maximum area of two-dimensional shapes that would fit into the PRSP volume, assuming these two-dimensional shapes would approximate to the three-dimensional case given the relatively negligible thickness. In our analysis of therapeutic dose of cells delivered discussed later in this manuscript, we assigned macroencapsulation devices a fixed maximum thickness of 0.6 mm to facilitate diffusion, and dimensions in the *x*- and *y*-directions are greater than one order of magnitude larger. The maximum-sized polygon with less than 10 sides was fitted into the left and right side of the linea alba where the median, maximum and minimum participants and resulting polygons are shown in figure 3*a*. The average area of these two polygons were analysed in figure 3*b–d* with respect to gender, age and BMI. Females were significantly smaller than males (male = 1.56 ± 0.52 × 10^4^ versus female = 1.367 ± 0.42 × 10^4^ mm^2^, ****p =< 0.001, figure 3*b*). Children were significantly smaller than young and older adults (0–18 = 1.139 ± 0.41 × 10^4^ versus 18–45 = 1.62 ± 0.37 × 10^4^ and >45 = 1.71 ± 0.47 × 10^4^ mm^2^, ****p < 0.001), while young adults were not significantly different to older adults, figure 3*c*. All BMI groups were significantly different to each other (<18.5 = 0.95 ± 0.31 × 10^4^ versus 18.5–24.9 = 1.39 ± 0.30 × 10^4^ versus 25–29.9 = 1.65 ± 0.39 × 10^4^ versus >30 = 1.85 ± 0.49 × 10^4^ mm^2^, ****p <= 0.001, figure 3*d*). There was a statistical difference in polygon area of each side (right = 1.49 ± 0.54 × 10^4^ versus left = 1.45 ± 0.52 × 10^4^ mm^2^, **p = 0.006, figure 3*e*).

### 2.3. Design of off-the-shelf devices to fit the PRSP

**Figure 4.**
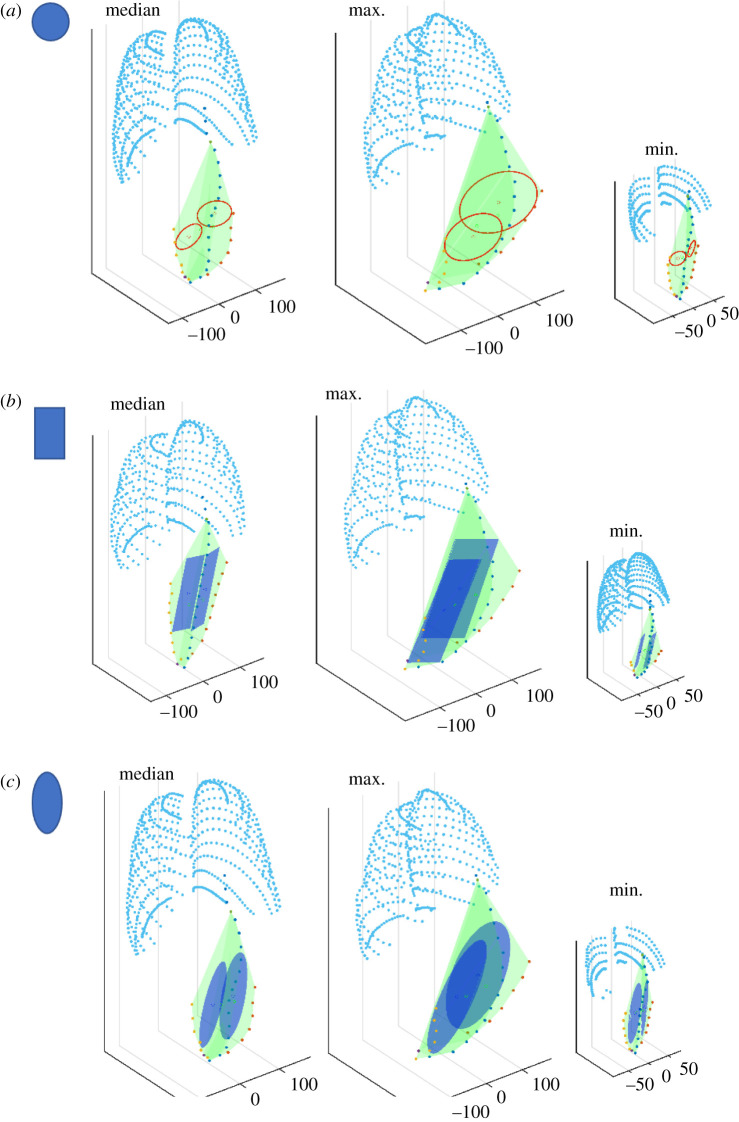
Graphical representation of the maximum fitted circular, rectangular and elliptical shaped devices in the PRSP. Graphical representation of the area of the maximum fitted circles (shown in red) (*a*), rectangles (shown in royal blue) (*b*), ellipses (shown in royal blue) (*c*), in the PRSP (shown in green) and ribs (shown in aqua) for the median, max and minimum participants.

For an off-the-shelf approach, uniform devices could be manufactured in several sizes. The maximum circular and rectangular devices, representing current devices under development, were fitted into the PRSP. We compared these results to a novel elliptical device that includes features of circular and rectangular devices, which we predicted would optimize the space. The median, maximum and minimum participants are shown in figure 4 for circular (figure 4*a*), rectangular (figure 4*b*) and elliptical devices (figure 4*c*).

**Figure 5.**
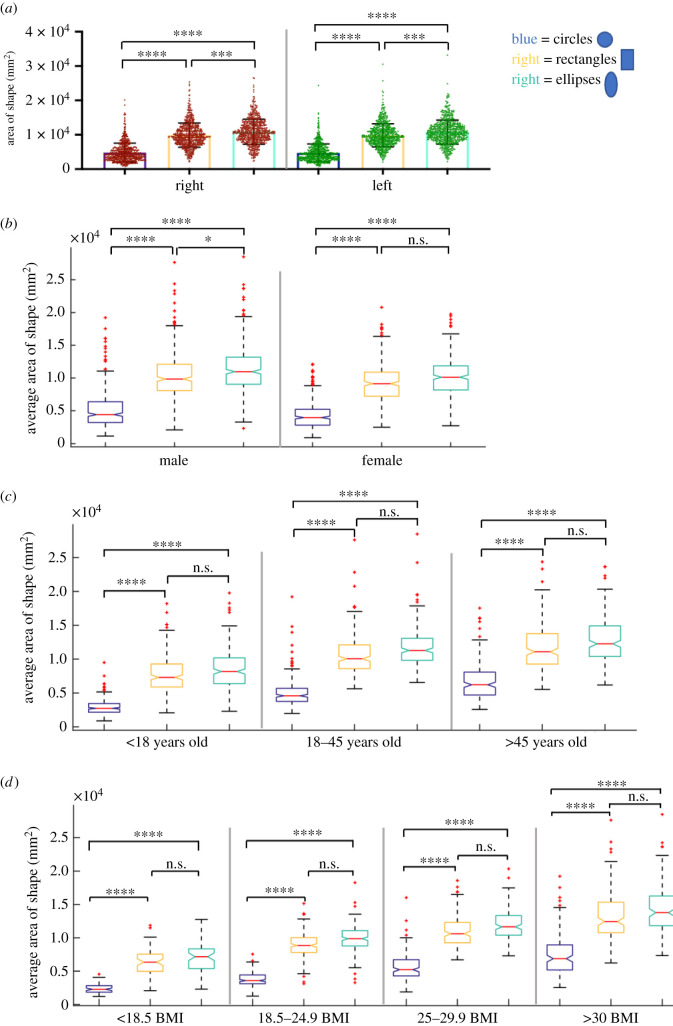
Analysis of the maximum fitted circular, rectangular and elliptical shaped devices in the PRSP. Analysis of the area of the maximum fitted circle, rectangle and ellipse on the left and right side of the linea alba (*a*). Analysis of the average area of the maximum fitted circle, rectangle and ellipse in the PRSP with respect to gender (*b*), age (*c*) and BMI (*d*). Data are means ± s.d.; *p < 0.05, ***p < 0.001, ****p < 0.0001, n.s., not significant.

We compared the area of the circular, rectangular and elliptical devices to each other on the left and right, figure 5*a*. Ellipses were significantly larger than circles and rectangles on both the left (ellipses = 1.08 ± 0.35 × 10^4^ versus circles = 0.47 ± 0.26 × 10^4^ ****p < 0.001, and rectangles = 0.98 ± 0.33 mm^2^ ***p < 0.001) and right (ellipses = 1.09 ± 0.36 × 10^4^ versus circles = 0.48 ± 0.27 × 10^4^ ****p < 0.001, and rectangles = 0.99 ± 0.96 × 10^4^ mm^2^ ***p < 0.001). Rectangles were significantly larger than circles on the left (****p < 0.001) and right (****p < 0.001).

The average area of these two shapes was analysed with respect to gender, age and BMI. In males, ellipses were significantly larger than circles and rectangles (ellipses = 1.14 ± 0.38 × 10^4^ versus circles = 0.51 ± 0.29 × 10^4^ ****p < 0.001, and rectangles = 1.03 ± 0.36 × 10^4^ mm^2^ *p < 0.027). Rectangles were significantly larger than circles (****p < 0.001). In females, ellipses were significantly larger than circles (ellipses = 1.02 ± 0.31 × 10^4^ versus circles = 0.44 ± 0.21 × 10^4^ mm^2^ ****p < 0.001), while there was no difference between rectangles and ellipses (rectangles = 0.93 ± 0.30 × 10^4^ versus ellipses = 1.02 ± 0.31 × 10^4^ mm^2^ *p* = 0.062). Rectangles were significantly larger than circles in females (****p < 0.001).

In all age groups, circles were consistently the smallest being statistically smaller than rectangles and ellipses (<18: circles = 0.29 ± 0.11 × 10^4^ versus rectangles = 0.76 ± 0.26 × 10^4^ and ellipses = 0.84 ± 0.29 × 10^4^ mm^2^ ****p < 0.001. 18–45: circles = 0.51 ± 0.21 × 10^4^ versus rectangles = 1.07 ± 0.29 × 10^4^ and ellipses = 1.18 ± 0.28 × 10^4^ mm^2^ ****p < 0.001. >45: circles = 0.68 ± 0.27 × 10^4^ versus rectangles = 1.17 ± 0.32 × 10^4^ and ellipses = 1.27 ± 0.32 × 10^4^ mm^2^ ****p < 0.001, figure 5*c*). There was no difference in rectangles and ellipses in any age category (<18: *p* = 0.787, 18–45: *p* = 0.164, >45: *p* > 0.999, figure 5*c*).

A similar trend was seen with BMI where circles were consistently the smallest being significantly smaller than rectangles and ellipses (<18.5: circles = 0.24 ± 0.08 × 10^4^ versus rectangles = 0.6373 ± 0.19 × 10^4^ and ellipses = 0.70 ± 0.22 × 10^4^ mm^2^ ****p < 0.001. 18.5–24.9: circles = 0.38 ± 0.10 × 10^4^ versus rectangles = 0.89 ± 0.186 × 10^4^ and ellipses = 0.99 ± 0.198 × 10^4^ mm^2^ ****p < 0.001. 25–29.9: circles = 0.56 ± 0.21 × 10^4^ versus rectangles = 1.09 ± 0.23 × 10^4^ and ellipses = 1.1975 ± 0.24 × 10^4^ mm^2^ ****p < 0.001. >30: circles = 0.74 ± 0.31 × 10^4^ versus rectangles = 1.32 ± 0.37 × 10^4^ and ellipses = 1.42 ± 0.35 × 10^4^ mm^2^, ****p < 0.001, figure 5*d*). There was no difference in rectangles and ellipses in any age category (<18.5: *p* > 0.999, 18.5–24.9: *p* = 0.198, 25–29.9: *p* = 0.783, >30: *p* > 0.999, figure 5*d*). Additional analysis of area of circles, rectangles and ellipses and aspect ratio of rectangles with respect to gender, age and BMI is in electronic supplementary material, figure S1, and comparing the right to left in electronic supplementary material, figure S2.

**Figure 6.**
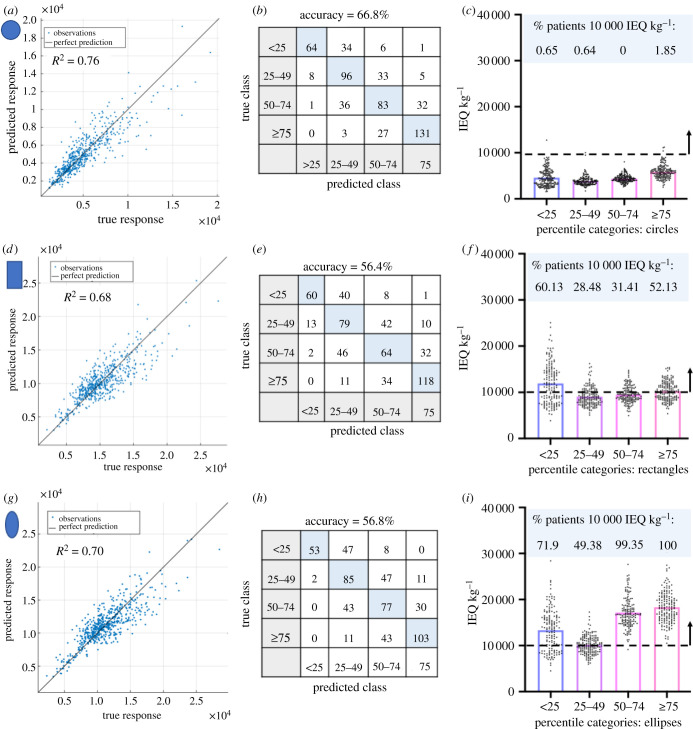
Interpretation of the maximum fitted circular, rectangular and elliptical shaped devices in the PRSP. Predicted versus actual plot for the observations using a quadratic support vector machine (SVM) regression model for each shape (*a*,*d*,*g*). Confusion matrix for the classification model for each shape where the class labels were defined as the percentile bands of the average area of the maximal fitted devices (less than 25th, 25–49th, 50–74th and greater than or equal to 75th percentile) (*b*,*e*,*h*). Number of cells (IEQ kg^−1^) that can be delivered using two devices based on the calculation that one IEQ corresponds to the tissue volume of a perfectly spherical islet with a diameter of 150 µm [39]. Thickness of 0.6 mm is assigned to devices and islet density of 10% of the volume fraction. The percentage of patients that achieve a therapeutic dose of 10 000 IEQ kg^−1^ is shown per percentile category for circular, rectangular and elliptical shaped devices in the PRSP (*c*,*f*,*i*).

We next sought to determine if anatomical parameters assessed during a routine physical examination could provide a screening tool to determine whether patients were anatomically suitable for a macroencapsulation device. A multivariate regression was performed for circular, rectangular and elliptical devices, with age, gender, BMI and the five anatomical distances as listed previously as the input features, and the average area of the maximal fitted devices for each patient as the response. The regression models were trained and tested using data from 560 patients, with any patient from the total 642 not having all eight attributes recorded excluded. A quadratic support vector machine (SVM) regression model trained and tested with 10-fold cross-validation [38] was found to give the best performance. For the circles the actual versus predicted plot in figure 6*a* shows *R*^2^ = 0.76. The root mean squared error (RMSE) was 1.295 × 10^4^ mm^2^ and the mean absolute error (MAE) was 0.90 × 10^4^ mm^2^. For the rectangles the actual versus predicted plot in figure 6*d* shows *R*^2^ = 0.68. The RMSE was 1.89 × 10^4^ mm^2^ and the MAE was 1.41 × 10^4^ mm^2^. Finally for the ellipses, the actual versus predicted plot in figure 6*g* shows *R*^2^ = 0.70. The RMSE was 1.87 × 10^4^ mm^2^ and the MAE 1.4 × 10^4^ mm^2^. Thus, the multivariate regression model is an adequate screening tool to estimate the maximum size device that can fit in the PRSP. Next, a classification analysis was performed using the same input features, to predict theoretical off-the-shelf devices for each shape. The classes were defined as the percentile bands of the average area of the maximal fitted devices (less than 25th, 25–49th, 50–74th and greater than or equal to 75th percentile), and training and testing with 10-fold cross-validation. The best performing model was a SVM with a medium Gaussian kernel giving an accuracy of 66.8% for circles (figure 6*b*), 56.4% for rectangles (figure 6*e*) and for ellipses a SVM with a coarse Gaussian kernel giving an accuracy of 56.8% (figure 6*h*). The receiver operating characteristic (ROC) curves are shown in electronic supplementary material, figure S3 for circles, rectangles and ellipses. Each shape has four corresponding ROC curves for each individual class compared to the other three. The classifier operating point is also shown. In all cases best performance is seen with the less than 25th and greater than or equal to 75th percentile classes with these ROC curves having an area under the curve nearer the ideal 1.0 compared to other classes. This provides an additional screening tool for physicians. Based on measurements obtained through a physical examination, we can estimate which device size would be most suitable to the anatomy. Patients deemed to have the appropriate anatomical criteria would undergo further imaging with a formal CT scan.

To interpret these results in terms of therapeutic dose of islets a thickness of 0.6 mm was assigned to give a volume. This thickness was chosen as cells should not be greater than 0.2–0.3 mm [25,40–42] from the porous membrane of immune isolation devices, where the ingrowth of vasculature is inhibited, to allow sufficient transport of vital oxygen and nutrients. Islet density has been recommended to be 5–10% of the volume fraction of the device [31] and we have used 10%. Based on these inputs we can deliver 33.97 IEQ mm^−3^. We determined the number of islets (IEQ kg^−1^) that could be delivered using two devices per patient, for each shape and each percentile category. We identified the percentage of patients within each category that could achieve a therapeutic islet dose (10 000 IEQ kg^−1^ [2,43,44]). For circular devices the percentage of participants that would achieve a therapeutic dose is 0.65% (4558 ± 1861 IEQ kg^−1^) <25th percentile, 0.64% (3888 ± 1018 IEQ kg^−1^) 25–49th percentile, 0% (4386 ± 823 IEQ kg^−1^) 50–74th percentile, 1.86% (45 850 ± 1460 IEQ kg^−1^) >75th percentile, figure 6*c*. For rectangles, 60.13% (11 921 ± 4313 IEQ kg^−1^) <25th percentile, 28.48% (9078 ± 2010 IEQ kg^−1^) 25–49th percentile, 31.41% (9504 ± 1765 IEQ kg^−1^) 50–74th percentile, 52.13% (10 250 ± 1991 IEQ kg^−1^) >75th percentile, figure 6*f*. For ellipses, 71.9% (13 337 ± 4456 IEQ kg^−1^) <25th percentile, 49.38% (10 141 ± 1956 IEQ kg^−1^) 25–49th percentile, 99.35% (17 164 ± 3203 IEQ kg^−1^) 50–74th percentile, 100% (18 313 ± 3386 IEQ kg^−1^) >75th percentile, figure 6*i*.

### 2.4. Polygonal and elliptical shaped macroencapsulation devices in PRSP can deliver a therapeutic dose of transplanted cells

**Figure 7.**
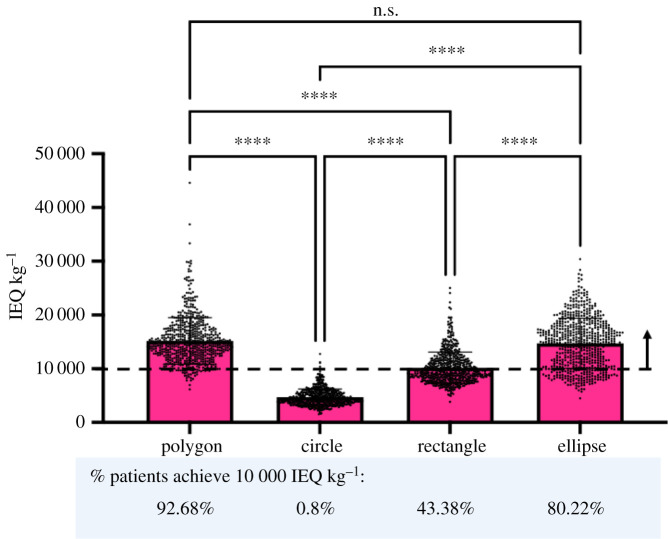
Comparison of polygonal, circular, rectangular and elliptical shaped devices in terms of therapeutic dose of cells (IEQ kg^−1^) that can be delivered in the PRSP. Number of cells (IEQ kg^−1^) that can be delivered using two devices based on the calculation that one IEQ corresponds to the tissue volume of a perfectly spherical islet with a diameter of 150 µm [39]. Thickness of 0.6 mm is assigned to devices and islet density of 10% of the volume fraction of the device. The percentage of patients that achieve a therapeutic dose of 10 000 IEQ kg^−1^ is shown for polygonal, circular, rectangular and elliptical shaped devices in the PRSP.

Using a similar analysis as described in the previous section, we next compared the number of cells that could be delivered with polygonal, circular, rectangular and elliptical shaped macroencapsulation devices across the representative patient population, figure 7. We found that polygons could deliver significantly more islets than circles and rectangles (polygon = 15 165 ± 4320 versus circles = 4682 ± 1532 and rectangles = 10 179 ± 2917 IEQ kg^−1^ ****p < 0.001). Rectangles and ellipses could deliver significantly more than circles (rectangles = 10 179 ± 2917 and ellipses = 14 725 ± 4667 versus circles = 4682 ± 1532 IEQ kg^−1^ ****p < 0.001), and ellipses could deliver more than rectangles (ellipses = 14 725 ± 4667 versus rectangles = 10 179 ± 2917 IEQ kg^−1^ ****p < 0.001). Importantly, there was no statistical difference between polygons and ellipses (*p* = 0.591). The mean of polygons, rectangles and ellipses was above 10 000 IEQ kg^−1^ where polygons could achieve the therapeutic dose in 92.68% of patients, circles in 0.8%, rectangles in 43.38% and ellipses in 80.22% of patients in the cohort analysed.

## 3. Discussion

In this study we analysed CT scans from 642 patients and have identified a potential extraperitoneal space with specific anatomical benefits (blood supply, oxygen, accessibility). We then used a shape optimization process to maximize the volume of macroencapsulation devices to deliver a clinically impactful dose of islets within this space. Although morphomics has been used to inform device design for other applications [45,46], no study has assessed implantation sites for macroencapsulation devices to this level of detail. A significant challenge of macroencapsulation devices is the large surface-to-volume ratio required to provide adequate diffusion of vitals factors into and out of the transplanted cells. Large devices are required to provide a therapeutic dose of transplanted cells and the optimal anatomical implant site is not yet known. Our approach would aid clinical trial design and potentially be an important preclinical step in the translation pathway when designing novel macroencapsulation devices.

We found females to be consistently statistically smaller than males. Although the prevalence of T1D in male and female populations do not differ drastically, research suggests the potential for much stronger risk factors for premature death and other serious complications in women [47,48]. Because of the differences in complications, risk factors and burdens associated with diabetes between genders, and acknowledging the longstanding gender bias in data collection and research, it is important for device designers to take gender factors into consideration. We also found statistical differences between groups with respect to age and BMI. Rates of overweight and obesity in T1D equal those of the general population [49], however, increased body weight and increased insulin demand are associated with more rapid disease progression after diagnosis of T1D in the 10–18 years age group [50]. We used these attributes, in addition with the series of five anatomical distances, as inputs for our regression models. Importantly these attributes and anatomical distances are readily categorizable and quantifiable non-invasively. It is envisioned such a characterization could be performed in a primary care setting. The outputs of these algorithms are a prediction of the average device size suitable for a given patient. Thus patients could be pre-screened for suitability for islet transplantation with a given device. A clinician could determine if a patient's anatomical parameters allow for an adequate islet dose to provide therapeutic efficacy. Patients with favourable anatomy would undergo a CT scan to verify the anatomical plane for operative planning, while those with unfavourable anatomy could pursue an alternative treatment strategy or undergo a supervised weight loss programme to reduce the therapeutic dose required. Here we use a regression model to predict specific sizes of devices. In all cases, the MAE for the predicted size was within 19% of the mean size for a given shape, with the elliptical shape being within 13%. This translates into an average error in the predicted area of the elliptical shape of 1400 mm^2^ which in therapeutic terms would be 95 000 IEQ. Classification analysis was also performed to investigate prediction of theoretical off-the-shelf devices for each shape using these same inputs with four sizes available in each case. The sizes were selected based on the minimum, 25th, 50th and 75th percentile sizes for a respective shape over the patient population. This defined four categories with a patient with a calculated device size less than the 25th percentile size ideally fitted with the minimum size, a patient between the 50th and 75th percentile with the 50th percentile size device and so on. While the overall general accuracy depending on the device type is between 56% and 67% it should be noted that classifiers performed well when considering the smallest or largest sizes in the range as shown by the ROC curves (electronic supplementary material, figure S3) indicating that with refinement and better threshold selection classification may also be an approach considered for screening purposes. It is also important to note that a random classifier would give an accuracy of 25%. While perhaps not currently fit for clinical use the promise of using such regression models and classification analysis with easy-to-measure input features has been demonstrated as a screening tool for patient stratification and pre-intervention planning prior to medical imaging. When we equated these device shapes and categories to therapeutic dose of islets that could be delivered to the PRSP, a thickness of 0.6 mm was assigned to the shapes to give a volume for a macroencapsulation device. We can determine the percentage of patients within each category who will achieve a therapeutic dose of 10 000 IEQ kg^−1^. For example, in a primary care setting our categorization analysis could predict that a patient will fall into the greater than or equal to 75th percentile category with 83% accuracy for the elliptical shaped device, and in our analysis, we found that 100% of the patients in this category achieved a therapeutic dose of 10 000 IEQ kg^−1^ with this shape of device. In addition, for this category, a further 8% of patients are misclassified as being in the 50–74th percentile category where 99.35% of patients achieve greater than 10 000 IEQ kg^−1^.

We found that polygon-shaped devices can achieve the largest area in the PRSP and as a result a significantly higher cell dose. The irregular nature a polygon shape makes this approach challenging to translate as a patient-specific device would be needed. The irregular shape makes them difficult to manufacture and may also result in higher stress concentrations at sharp angles that have been shown to induce a strong foreign body response [32]. The significant difference between the left and right sides for polygons adds complexity when planning the procedure. We have, recently, described a porous refillable reservoir and a minimally invasive procedure to deliver it between muscle layers in a pig study, with securement to the fascia via a minimally invasive scope and trocar system [51]. We have shown that a space can be created, and a reservoir (similar size as discussed here) deployed in an atraumatic manner and secured with current minimally invasive fixation tools. These refillable reservoirs are manufactured from soft materials such as thermoplastic polyurethane, with an elastic modulus approximately 15 MPa [52], similar to the tissue extracellular matrix [53], which enables minimally invasively delivery through a trocar [51] and also allows conformation of the device during bending and twisting within the tissue plane. This favourable interaction with the surrounding environment will be essential for long-term device performance but also patient comfort. However, long-term performance of these devices in a diabetic pig model has not yet been tested. Additionally, patient comfort is an important consideration with devices of this size and shape and this metric would need to be tested in a clinical trial setting. This material also has excellent manufacturing flexibility where reservoir shape, size and volume can be easily modified [51,52,54,55]. Additionally, an inner support structure can be easily added to support dispersion of the islets throughout the device and prevent islet clumping [51,56,57]. We have used a laser cutting technique to generate pores in the reservoirs where pore size, pattern and density can be varied depending on the specific clinical need [51,52], while pores less than 10 µm provide an immune-protective barrier (via size exclusion of immune cells) but still allow diffusion of macromolecules and paracrine factors. This technology was designed and manufactured for use in a large animal model that are easily translated to human studies, and we demonstrated clinical validity through delivery and deployment to the abdominal wall of a pig, and in doing so exploited existing surgical techniques for abdominal wall reconstruction [51]. We envisage that this technique can be used for refillable macroencapsulation devices delivered to the PRSP. However, from this work, we have learnt the challenges of delivering an irregular shaped device in a minimally invasive manner, particularly for deploying a device from a trocar to a flat position for securing to underlying fascia. Polygon-shaped devices have potential for use if the volume of payload to be delivered is prioritized, where a patient-specific device could be manufactured after imaging of the patient and delivered surgically. More uniform symmetrical shapes like circles, rectangles and ellipses are easier to manufacture and deliver minimally invasively. Interestingly, there was no significant difference between the number of cells that could be delivered with the polygon and elliptical shaped devices, where the percentage of the cohort of patients analysed can achieve greater than 10 000 IEQ kg^−1^ was 92.68% for polygon and 80.22% for elliptical shaped devices. In our analysis, both circular and rectangular-shaped devices fall short of this number, where 0.8% and 43.38% achieve this therapeutic dose of 10 000 IEQ kg^−1^. Remarkably, macroencapsulation devices in these shapes are in the most advanced stages of clinical development. We found that circular devices could deliver the lowest cell number (4682 ± 1532 IEQ kg^−1^) and interestingly Beta-O_2_ Technologies have reported delivering 1800–4600 IEQ kg^−1^ with their circular β-Air device [15]. It must be noted that β-Air includes an oxygenation strategy, so they may not be limited to thickness or volume fraction that we have assigned so can potentially achieve a higher cell payload with their device. Other circular macroencapsulation devices currently in development may potentially be faced by this challenge. However, here, we have described a scenario where one large device is used but more than one device could potentially be stacked on top of each other, allowing vascular ingrowth between the stacked devices, to increase this delivered volume even further. Nonetheless, in our analysis, we found that elliptical shaped devices meet manufacture and delivery criteria, such as uniform shape and no sharp edges, while also achieving a therapeutic dose of greater than 10 000 IEQ kg^−1^ in 80% of the patient cohort analysed in this study.

There are some limitations with the current study. The machine-learning element of this work will need to be validated firstly in a human cadaver model, while efficacy of our strategy to reverse T1D will need to be tested in a diabetic pig model. We have established a streptozotocin (STZ)-induced diabetic pig model with our collaborators [56] and the morphomics approach we describe in this study can be easily used to quantify the PRSP in a pig model from CT scans. The MATLAB segmentation was based on the cartesian coordinates in *R*^3^ of anatomical landmark points for each patient including the xyphoid caudal tip, pubic symphysis cranial tip and then at 30 mm intervals in the craniocaudal direction the position of the linea alba and left and right semilunar points. The left and right PRSPs as defined by Delaunay triangulation of these points are hence an approximation of the true anatomy with the computational models interpolating between the points. The maximal fitting shapes were calculated in cutting planes taken parallel to the dorsal to ventral most faces of the minimally fitting bounding box calculated around each PRSP. This approach was taken to give the series of parallel frontal sections of greatest area for a given PRSP, with the assumption the largest fitting shape for the volume lies in one of these planes. While it is accepted there may exist a two-dimensional plane in a different orientation in three-dimensional space that may fit a larger shape then that calculated this is both a computationally challenging problem to solve and may not be of clinical value.

We have identified the PRSP as a potential implant site for macroencapsulation devices as it meets the requirements for being easily accessible, can facilitate longitudinal monitoring of transplants and can provide nutritive support for cell survival. We have analysed this space using morphomics across a patient cohort and have analysed the data in terms of gender, age and BMI. We used a shape optimization process to maximize the volume of macroencapsulation devices to deliver a clinically impactful dose of islets within this space. We have identified that elliptical shaped devices can achieve a clinically impactful cell dose while also meeting device manufacture and delivery requirements. This information has the potential to significantly impact the field and may influence the design of future macroencapsulation devices to better suit the needs of patients with T1D.

## 4. Methods

### 4.1. Study cohort

We performed a retrospective analysis of patients who underwent CT scans at the University of Michigan as part of evaluation for kidney donation between years 2002 and 2015. This study cohort has previously been used as a healthy reference population [58,59]. Patient age, sex, height and weight were obtained from their medical record prior to evaluation for kidney donation. Patients were included if they had a non-contrast-enhanced series CT scan performed as part of evaluation for kidney donation, with a complete fascia boundary visible in the display field of view, had age, sex, height and weight recorded in their electronic medical record, and were medically, surgically and psycho-socially approved for donation. Body mass index was computed and categorized into groups according to the World Health Organization. CT imaging was extracted for 642 total patients. Patient scans were obtained using the GE ‘Standard’ reconstruction algorithm at 120 kVp and up to 5 mm slice thickness in a Discovery or LightSpeed scanner. Tube current was automatically modulated to tissue radio-density within each axial body cross-section.

### 4.2. Segmentation and MATLAB analysis

The CT scan data recorded the positions in *R*^3^ of the caudal most point of the xiphoid process, the cranial tip of the pubic symphysis, the linea alba (central blue marker in figure 1*b*) and the left (orange) and right (yellow) semilunar points (lateral markers in figure 1*b*) at each level. The linea alba and left and right semilunar points were recorded at approximately 30 mm intervals in the craniocaudal direction.

The PRSP was defined bilaterally as the space enclosed by the xiphoid, pubic symphysis, linea alba and the semilunar points. Using MATLAB, the Delaunay triangulation was computed of this space on both the left and right sides using the defined boundary points. The surface produced was taken as the boundary of the left and right PRSPs which enclosed a volume. These geometries were computed in a similar manner for each patient.

To calculate the best fitting devices a series of slices were taken at 1 mm intervals across each of the volumes, with the maximally fitting circle, rectangle and ellipse calculated in each slice. The largest of these respective shapes across all the slices for that volume was taken as the maximally fitting circular, rectangular or elliptical shape for that PRSP. Additionally, the largest slice in terms of area was taken as the largest polygon for that volume. This was done on both sides and for all patients, with metrics calculated on the maximally fitting shapes including area, centre of mass and aspect ratio in the case of the rectangle. The slicing planes were taken as the set of planes running parallel from the dorsal to ventral most faces of the minimally fitting bounding box calculated around each PRSP.

### 4.3. Statistics

Graphpad Prism was used for statistical analysis. Normality was tested with a Shapiro–Wilk test. All data were not normally distributed. A Kruskal–Wallis test was used with Dunn's post hoc adjustment for multiple comparisons was used. In the case of paired data Wilcoxon matched pairs signed-rank test was used. Statistical significance was accepted when *p* < 0.05.

## Data accessibility

Raw data available at https://datadryad.org/stash/share/XGLbN46Jc_RANmvLJxC36cLi4XX4VMxT89mdd5qOnQs.
